# Pyoderma Gangrenosum, a Challenging Postpartum Diagnosis—Case Report and Literature Review

**DOI:** 10.3390/jcm13133653

**Published:** 2024-06-22

**Authors:** Daniela Roxana Matasariu, Iuliana Elena Bujor, Elena Mihălceanu, Tudor Cătălin Gîscă, Alina Stâncanu, Elena Corina Andriescu, Ioana Popescu, Demetra Socolov, Ciprian Vasiluță, Alexandra Ursache

**Affiliations:** 1 Department of Obstetrics and Gynecology, University of Medicine and Pharmacy “Gr. T. Popa”, 700115 Iasi, Romania; daniela.matasariu@umfiasi.ro (D.R.M.); gisca_tudor-catalin@d.umfiasi.ro (T.C.G.); demetra.socolov@umfiasi.ro (D.S.); alexandra.ursache@umfiasi.ro (A.U.); 2 Department of Obstetrics and Gynecology, Cuza Voda Hospital, 700038 Iasi, Romania; 3 Department of Dermatovenerology, “Saint Spiridon” County Hospital, 700111 Iasi, Romania; emaldacalin@yahoo.com (A.S.); oana.manolache@yahoo.com (I.P.); 4 Department of Pathology, “Saint Spiridon” County Hospital, 700111 Iasi, Romania; andriescu_corina@yahoo.co.uk; 5 Department of Dermatology, University of Medicine and Pharmacy “Grigore T. Popa”, 700115 Iasi, Romania; 6 2nd Department of Surgery, “Saint Spiridon” County Hospital, 700111 Iasi, Romania; vascip2580@gmail.com

**Keywords:** pyoderma gangrenosum, postpartum, cesarian section, vaginal delivery, skin, histopathology

## Abstract

**Background/Objectives**: The infrequent occurrence of pyoderma gangrenosum (PG) during pregnancy and in postpartum, with its subsequent diagnostic intricacies, caused us to present the following case. **Methods**: This article describes a rare case of PG in postpartum in a patient without any prior pathology and a short review of the literature, aiming to identify similar rare instances. **Results**: We conducted a literature review to ascertain the prevalence of postpartum pyoderma gangrenosum, and we identified a total of 41 cases. **Conclusions**: Our article underlines again the importance of interdisciplinary collaboration for the prompt identification and commencement of necessary therapeutic interventions in postpartum women afflicted by pyoderma gangrenosum.

## 1. Introduction

Pyoderma gangrenosum (PG) represents a rare and intricate noninfectious inflammatory neutrophilic dermatosis, associated with substantial morbidity [1,2,3]. Originating from the initial description by Brunsting et al. in 1930, this dermatosis predominantly afflicts females, typically within the age range of 20 to 50 years [1,2,4,5,6]. The typical anatomical targets of PG encompass the lower limbs, hands, feet, and trunk, with the occasional involvement of diverse sites such as the genital mucosa, eyes, spleen, and lungs [1,7]. Distinct clinical variants of PG include the classic ulcerative type and another three forms (vegetative, bullous–pustular, and superficial granulomatous) [1,7]. The bullous subtype is frequently associated with hematologic disorders such as myelodysplastic syndrome [3,8,9].

While historically perceived as idiopathic with an incompletely understood etiology, PG demonstrates strong associations, observed in up to 50% of cases, with inflammatory and autoimmune conditions such as Crohn’s disease, inflammatory bowel disease (e.g., ulcerative colitis), monoclonal gammopathies, hematological malignancies, and rheumatoid arthritis. Other associated conditions are pregnancy, seronegative arthritis, viral hepatitis, and HIV [2,10,11]. Originally hypothesized to have an infectious origin due to symptomatic similarities with bacterial infections, recent research underscores an underlying inflammatory and autoimmune pathophysiology. This is characterized by sterile neutrophilic infiltrates devoid of fibrinoid necrosis in blood vessels, as corroborated by Ahn and Gameiro [2,11]. Additionally, PG is susceptible to complications from secondary bacterial infections, as emphasized by Suzuki.

The aberrant immune response in afflicted individuals typically commences with the formation of an erythematous papule, pustule, or vesicle, progressing swiftly to the development of chronic, recurrent, and painful necrotic ulcers surrounded by a violaceous hue [1,10,12,13]. Early lesions show neutrophilic folliculitis/perfolliculitis with dermal abscess. Later lesions are ulcerated with mixed dermal inflammation and neutrophilic abscess that undermine the ulcer edge. These ulcers may advance to disfiguring cribriform scars [1,3]. Nevertheless, other ulcerative conditions may present with a similar appearance, engendering potential diagnostic inaccuracies, as articulated by Park [14]. The underlying etiology remains elusive, although neutrophil dysfunction, the release of inflammatory mediators, or genetic predisposition may be implicated. The intricate pathogenesis of PG involves various precipitating factors and the characteristic phenomenon of pathergy, whereby distinct lesions emerge or pre-existing ones worsen following trauma [2,4,15]. Noteworthy risk factors include physical trauma, specific medications, and the external compression of the skin, such as those involving seat belt compression [2,16,17]. Recent investigations underscore the role of prolonged external compression and internal vascular compression, shedding light on diverse triggers for this enigmatic dermatological disorder [2,16].

The infrequent occurrence of PG during pregnancy and the postpartum period, although exceedingly rare, provides supplementary backing for the hypothesis of an inflammatory and immunosuppressive origin [4,18,19]. Despite its exceptional rarity, the diagnostic intricacies associated with PG are exacerbated, particularly in these specific clinical scenarios.

This article presents a rare case of PG in postpartum, in a patient without any prior pathology. By describing it, we aim to increase the awareness and comprehension of this intricate dermatosis, underscoring the significance of a nuanced diagnostic approach and collaborative efforts across different medical disciplines for effective management.

## 2. Case Report

### 2.1. Patient History and Physical Examination

A 28-year-old patient at 40 weeks of amenorrhea, under the care of a specialist in obstetrics and gynecology, was admitted to our department due to painful uterine contractions in preparation for childbirth. The patient had no significant personal medical history aside from a previous elective abortion in 2016, and she had an OI blood type with Rh negative. Blood, urine, and vaginal secretion samples were collected for analysis. These tests revealed a hemoglobin level of 11.8 mg/dL with a normal hematocrit and a white blood cell count (WBC) of 12.95 × 10^3^/μL, with a slightly elevated neutrophil count (10.26 × 10^3^/μL—79.2%).

Initially, a vaginal delivery was attempted since the fetus was in a cephalic presentation and had an estimated ultrasound weight of 3000 g. However, a decision was made to proceed with a cesarean section (C-section) due to the diagnosis of acute fetal distress, fetal bradycardia, and the presence of meconium-stained amniotic fluid on 11 September 2023. The result was a live male newborn, weighing 2910 g, with an Apgar score of 7.

Postoperatively, the patient was placed on antibiotic therapy with third-generation cephalosporin due to the extended interval of more than 6 h between the rupture of membranes and the prophylaxis of thromboembolism with a low-molecular-weight heparin at 40 mg/day (LMWN). Postoperatively, the patient presented a hemoglobin level of 11 mg/dL and an increased neutrophil count (19.79 × 10^3^/μL—91.6%). The patient was discharged 72 h after the cesarean section, with the surgical wound in the process of healing, in good general condition, without subjective complaints. She was advised to continue antibiotic treatment for up to 7 days postoperatively and to return for suture removal.

**Figure 1:**
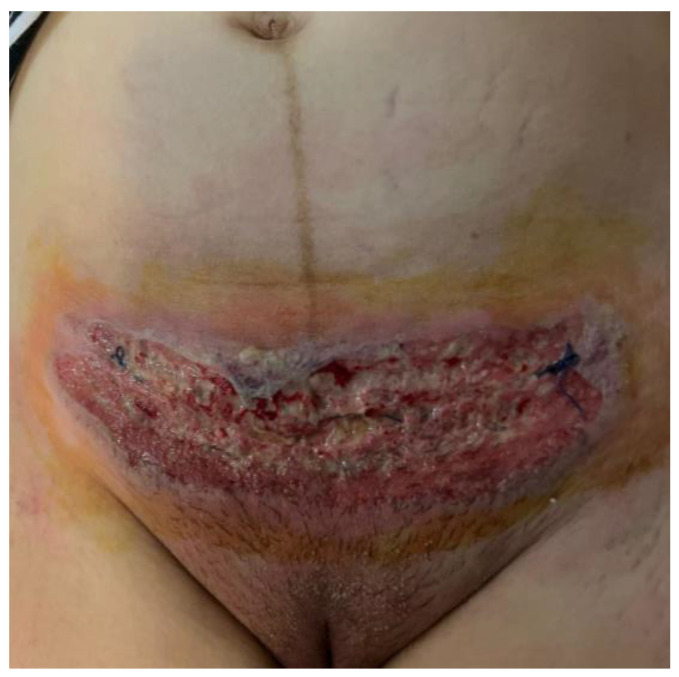
Patient surgical wound after cesarean section—day 7.

On the 7th day after surgery, the patient presented to the surgical department of another hospital, with localized symptoms at the wound site suggestive of superinfection (Figure 1). Without any significant prior personal history of the patient, the surgeons were sure that it was a surgical site infection so they decided to restart antibiotherapy in association, and only after they saw the aggravation of the lesion, and the fact that it was unresponsive to antibiotherapy and the cultures were negative they sent the patient for a dermatological evaluation.

The local examination objectifies an extensive erythematous-purulent ulcerative plaque with centrifugal development, relatively well-defined with irregular contour, tending to spread superficially. There were intensely painful purulent secretions located at the site of the post-cesarean scar. The edges of the lesion are ragged, undercut, and violaceus.

### 2.2. Histopathology Findings

**Figure 2:**
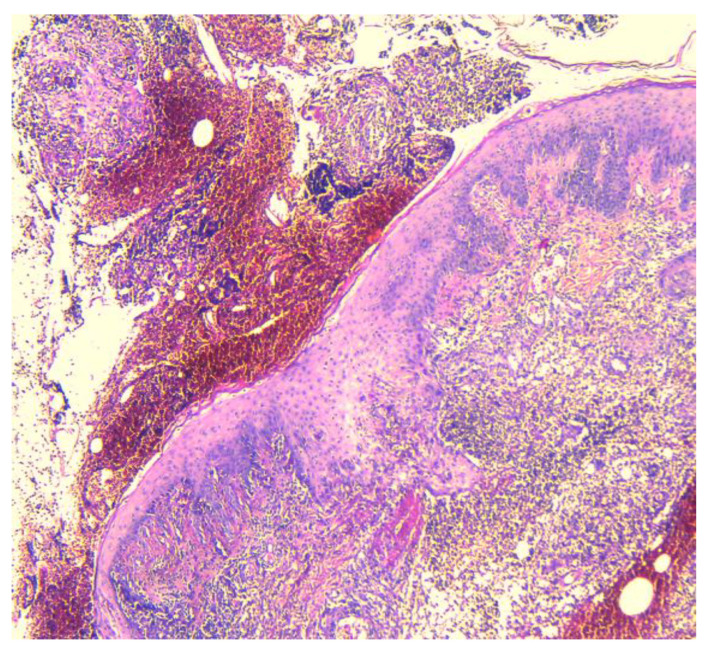
Ulcerative pyoderma gangrenosum—fibrino-hemato-leukocyte deposits, acanthosis, and pseudoepitheliomatous hyperplasia in the immediate vicinity of the ulcerated areas (50×, HE).

**Figure 3:**
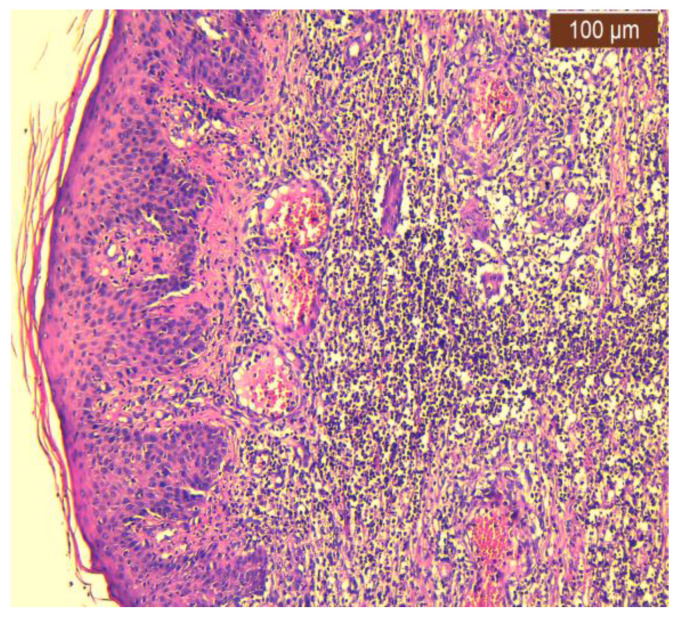
Ulcerative pyoderma gangrenosum—polymorphic inflammatory infiltrate throughout the thickness of the dermis with a perivascular disposition and the dissociation of collagen fibers (100×, HE).

**Figure 4:**
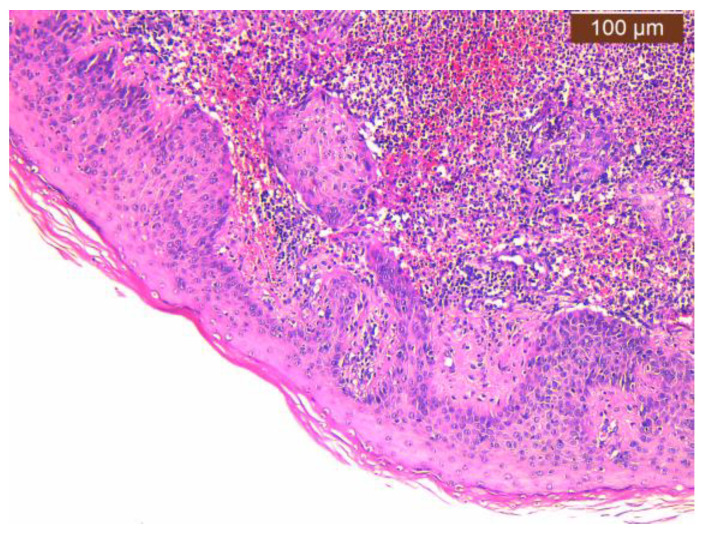
Ulcerative pyoderma gangrenosum—polymorphic inflammatory infiltrate throughout the thickness of the dermis, acanthosis, and pseudoepitheliomatous hyperplasia in the vicinity of the ulcerated areas (100×, HE).

In our case, areas of ulceration are identified with rich polymorphic inflammatory infiltrate in the thickness of the dermis and zonally of the hypodermis, with a tendency to abscess for formation. We also noted marked edema, leukocytoclastic vasculitis, and acanthosis with pseudoepitheliomatous hyperplasia in the perilesional zone (Figure 2, Figure 3 and Figure 4).

### 2.3. Dermatological Management

**Figure 5:**
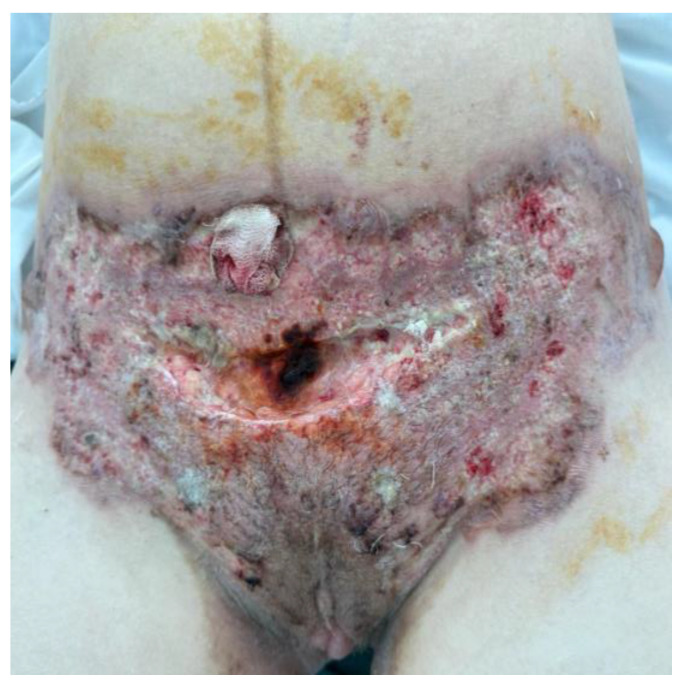
Wound aspect under antibiotherapy—day 16.

On the 16th day after cesarean section, the patient was referred to the Dermatovenereology Clinic of “Sfântul Spiridon” Hospital in Iași, transferred from the General Surgery Clinic II, due to the appearance and persistence of a relatively well-defined ulcerative plaque with purulent secretions on the surface, which were intensely painful, appearing at the cesarean scar approximately 1 week postoperation. It is noteworthy that the patient underwent intravenous treatment with antibiotics (a mixture of Lincosamides and Ureidopenicillins), analgesics, antipyretics, and anticoagulants (a low-molecular-weight heparin) and underwent a skin biopsy (pyoderma gangrenosum) from the lower abdominal lesion at the General Surgery II department (Figure 5).

Upon admission, the patient was afebrile, with good appetite, presented a slightly influenced general condition, and was hemodynamically and respiratorily stable. Abdominal examination reveals mobility with respiratory movements; however, palpation cannot be performed due to local abdominal pain. The patient reports normal bowel movements and physiological urination, and there are no signs of meningeal irritation. At the time of admission, the patient weighed 61 kg.

Local examination reveals an extensively ulcerative plaque laterally spreading that was centrifugal and erythematous-purulent, relatively well-defined with irregular borders, and tending to spread superficially, with intensely painful purulent secretions located at the post-cesarean scar.

Paraclinically, an inflammatory syndrome is observed (elevated CRP), including leukocytosis with neutrophilia, normochromic normocytic anemia, thrombocytosis, increased serum ferritin levels, altered electrolyte levels (hypokalemia, hyperchloremia), metabolic acidosis (serum bicarbonate—19.6 mmol/L), mild hypoproteinemia, hypocalcemia, and pathologic urine analysis (glucosuria+, microscopic hematuria, leukocyturia). These values normalize dynamically under instituted systemic and topical therapy. The bacteriological examination of secretions collected from the wound site was negative.

During hospitalization, systemic antibiotic therapy was administered (Lincosamides 600 mg every 8 h and Ureidopenicillin 4.5 g every 6 h intravenously) for 14 days, along with probiotics, analgesics, antipyretics, anticoagulants, and intravenous corticosteroid therapy for 6 days followed by oral corticosteroids 56 mg/day, along with associated corticosteroid medication (calcium gluconate, proton pump inhibitor, KCL), antifungals (150 mg—2 tablets/day), and the topical application of sterile neutral ointment dressings to the ulcerations, followed by re-epithelializing ointment, with favorable symptomatic evolution.

The patient’s progress was slowly favorable, and she was discharged with the following recommendations: hygienic–dietary regimen with salt avoidance, the continuation of oral corticosteroids (54 mg/day, with gradual dose reduction) along with associated corticosteroid medication and analgesics as needed, the topical application of the re-epithelializing ointment with a topical corticosteroid to the ulcerations until complete re-epithelialization, and coverage with sterile neutral ointment dressings.

**Figure 6:**
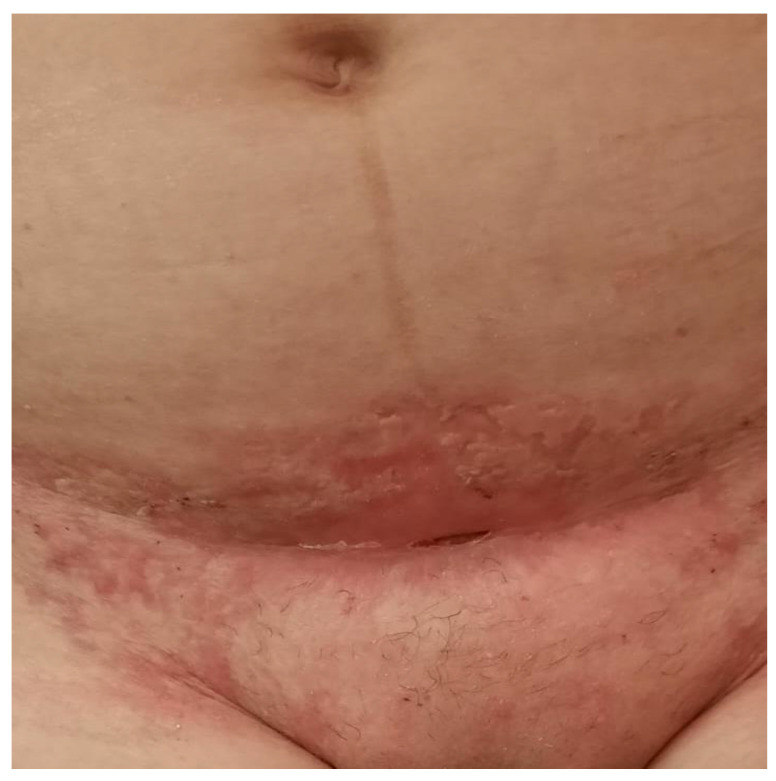
Patient incision after specific treatment—two months.

The patient’s progress was favorable but prolonged, with complete re-epithelialization of the skin ulcers occurring over two months (Figure 6).

## 3. Literature Search—Methodology and Results

We conducted a literature review to ascertain the prevalence of postpartum PG cases, identifying a total of 41 instances [4,5,6,10,14,15,18,20,21,22,23,24,25,26,27,28,29,30,31,32,33,34,35,36,37,38,39,40,41,42,43,44,45,46,47,48,49,50,51,52,53], including two documented in posters [35,53]. Among these cases, there is one that reported two occurrences of PG [42], while the remaining authors presented singular instances (Table 1).

## 4. Discussion

Pyoderma gangrenosum (PG) is a rare neutrophilic non-infectious dermatosis that is usually identified using exclusionary criteria [1]. Almost half of the patients suffering from this disease usually have concurrent comorbidities like inflammatory bowel disease, hematological diseases, and arthritis [6,8,10]. There are several forms of PG; the ulcerative (classical) form accounts for around 85% of cases, while the other types—bulbous, pustular, and vegetative—are linked to particular ailments.

According to George C et al., the prevalence of PG in the general population is estimated to be roughly 0.63 per 100,000 individuals, whereas Rao et al. observed 3–10 cases per million people annually [3,6]. Its frequency during pregnancy is yet unknown, nevertheless [6,54]. Although the precise cause of PG remains unknown, it is known to be either idiopathic or linked to systemic illnesses such as inflammatory bowel disease and other autoimmune disorders [1,14].

Pathergy is a typical observation in PG patients, which is an increased skin response to small trauma, including pressure, physical trauma, or needle stick injuries [1,14]. Rashid and Keskin et al. have reported cases where PG was caused by external factors, such as compression from a seat belt during a car ride or minor blunt trauma in a battered child, whereas Sarwar et al. presented a case in which internal skin compression from breast engorgement acted as a causative factor [1,17,55].

Pregnancy and the postpartum period have been identified as significant triggers for PG, with reports of postpartum cases often complicating cesarean section incision wounds [5,14,19]. Cokan et al. reported a case of PG developing in the breast eight weeks postpartum following cesarean section PG. The immunosuppressive effect of pregnancy is posited as a potential explanation, as discussed by Cokan [36].

PG, characterized by autoinflammatory features involving both innate and adaptive immune systems and underlain by a genetic predisposition, results in abnormal neutrophil deposition and elevated levels of inflammatory mediators such as interleukin (IL) 1β, IL-8, IL-17, and tumor necrosis factor α. The precise mechanism linking pregnancy and PG remains elusive, with pregnancy associated with progressive neutrophilia and an inflammatory cascade during labor. Postpartum PG is infrequent and predominantly manifests at cesarean section incision sites [4,5,14,53,56]. Although the exact relationship between the inflammatory pathways in pregnancy and postpartum PG is unknown, a change in Th1 and Th17 expression may be a shared cause. Th1 becomes more dominant towards the end of pregnancy, which supports Th17 cytokines’ involvement in the pathophysiology of PG [19].

The absence of universally accepted diagnostic criteria for PG poses a real clinical challenge. Su et al. designated ulcerative PG as a diagnosis of exclusion, which may prove impractical and hinder diagnosis [7]. A more recent approach, validated by Maverakis, involves considering one major criterion (biopsy at the ulcer edge demonstrating a neutrophilic infiltrate) and at least four of eight minor criteria for diagnosis, demonstrating high sensitivity and specificity [57]. The eight minor criteria include the following: exclusion of site infection; the presence of the pathergy phenomenon; personal history of inflammatory bowel disease or inflammatory arthritis; personal history of rapid ulceration of papules, pustules, or vesicles; peripheral erythema with undermining border and tenderness at the lesion site; multiple ulcerations, with at least one of them on an anterior lower leg; scars at healed lesion sites; and decreased ulcer size after one month of initiating immunosuppressive medication(s) [57].

To support the diagnosis, bacterial, mycobacterial, and fungal tissue cultures should be obtained to exclude infectious etiology. Additionally, laboratory studies, such as erythrocyte sedimentation rate and C-reactive protein, although lacking specificity, may be considered. Given the association of PG with Crohn’s disease or ulcerative colitis in approximately 50% of cases, a comprehensive gastrointestinal review, along with a screening fecal calprotectin serologic test, is recommended. Evaluation for a history of hidradenitis suppurativa is advisable. Approximately 10% of cases are associated with leukemia; hence, assessment for a monoclonal gammopathy, including protein and urine electrophoresis and Bence Jones protein analysis, is suggested [56]. Hepatitis, syphilis, and HIV testing are reasonable considerations. Since 30% of patients exhibit coexisting arthritis, evaluation with rheumatoid factor and anti-cyclic citrullinated peptide is also warranted. Furthermore, if indicated by a thorough review of systems, a hypercoagulability work-up should be considered [3,56,58].

The differential diagnosis of skin ulcerations encompasses various etiologies, including primary infections (bacterial, fungal, and viral), vasculitis, malignancy, vascular occlusive or venous diseases, drug-induced or exogenous tissue injury, and other inflammatory disorders [59]. Pertinently, instances of pyoderma gangrenosum (PG) are frequently misidentified as wound infections, leading to potential delays in diagnosis and treatment. The initial presentation often lacks severity, manifesting only as pain, erythema, and engorgement [6,10]. Moreover, a variant of Sweet syndrome, known as bullous Sweet syndrome, is commonly identified in individuals with leukemia that presents overlapping symptoms [56].

A comprehensive analysis of the existing literature pertaining to the treatment of pyoderma gangrenosum (PG) reveals that the primary therapeutic approach involves the administration of immunosuppressants in conjunction with local wound care. It is imperative to counsel patients with a history of PG on the potential exacerbation of lesions due to compression or blunt trauma [1,60].

The preferred treatment modality entails systemic high-dose corticosteroids, with successful outcomes reported with various immunosuppressive agents [6]. Topical interventions demonstrate heightened efficacy in superficial PG cases [5]. For small lesions lacking systemic symptoms, initial therapy involves topical anti-inflammatories such as corticosteroids, tacrolimus, and dapsone. Larger wounds may necessitate systemic therapy, including high-dose corticosteroids or immune modulators, if topical regimens prove insufficient [6,14,58]. Glucocorticoids, particularly prednisone, are typically administered at high doses until all lesions heal, followed by lower maintenance doses to prevent recurrence. Alternatives such as systemic cyclosporine, dapsone, and minocycline are viable options [6,58]. Biologics, specifically from the tumor necrosis factor α class (e.g., infliximab and adalimumab), are emerging as first-line therapies due to their rapid response, tolerable adverse effect profiles, and finite duration of use [14,58].

It is recommended to avoid staples and to use sutures for the incision closure and to take into consideration the use of preoperative steroid administration to minimize the risk of pathergy. In cases where lesions develop, the initial therapeutic approach involves optimizing wound care, minimizing pathergy from additional surgical intervention, and initiating topical or intralesional therapy as delineated below [58].

Prophylactic perioperative corticosteroids are recommended for preventing postoperative PG in predisposed patients undergoing planned surgery. In emergency surgeries, the immediate initiation of this treatment post-surgery is advised [14].

Autologous skin grafting is a consideration for extensive lesions [61], but the potential for disease flare-up at the donor site exists [14]. Case reports, such as that by Goto et al., have documented the successful treatment of PG through a combination therapy involving oral prednisolone, vacuum-assisted closure (VAC), and skin grafting [62]. Additionally, the application of skin grafting and/or negative pressure wound therapy has exhibited promise in small-scale studies [53]. Hyperbaric oxygen therapy can be employed to further enhance wound healing [4].

Notably, in 37 cases, the lesions originated at the C-section site, but sometimes with additional regions affected in select cases [4,10,14,15,18,20,21,22,23,24,25,26,27,28,29,30,31,32,33,34,35,36,37,38,39,40,41,42,43,44,45,46,47,48,49,50,51,52]. For instance, Sanz-Munoz et al. observed lesions on the anterior side of the left leg prenatally [5], Wierzbicka-Hainaut noted lesions on the left calf [30], Gunduz reported bilateral gluteal regions post-injection [45], and Cokan associated lesions with the breast region [36]. Other variations in lesion presentation included Futami et al. reporting lesions on the face, neck, upper arm, and thigh post-vaginal birth [23], Rao et al. documenting lesions at the episiotomy site [6], and Lyons et al. observing lesions in the breast region [53]. From the cases included in this study, it was observed that Futami and Rao et al. reported instances of vaginal childbirth, while Aoussar and Lyons et al. did not specify the mode of childbirth in their cases [6,23,27,53].

Roa and Futami et al. presented cases of PG in patients who gave birth vaginally, while the means of birth were unspecified in Aoussar et al. and Lyons et al. [6,23,27,53]. Premature birth occurred in 13 cases due to various emergencies, such as chorioamnionitis, abruptio placenta, and vaginal bleeding [10,20,21,33,35,37,40,42,43,44,47,48,51]. However, the method of delivery was unspecified in 16 cases [4,5,6,18,24,27,28,29,31,36,38,39,45,46,49,52], with the remaining cases delivering at term. Notably, 15 cases of postpartum C-section site PG were recurrent [15,24,25,27,30,32,33,34,40,41,42,43,46,48,52], only 1 case exhibited the first episode of the disease linked to surgical intervention with subsequent relapses [23], and in the rest of the included cases, this was the first manifestation of the disease.

Karim et al. presented a case with a familial history of pyoderma gangrenosum, with the patient’s sister also suffering from the disease [26]. Associated pathologies identified included ulcerative colitis [23], chronic type C hepatitis [24,38], Takayasu arteritis and type B hepatitis [27], weakly positive antiphospholipid antigen [14], antiphospholipid syndrome with weakly positive lupus anticoagulant and three spontaneous abortions [37], type B hepatitis [49], thrombocytopenia early in pregnancy [51], and a fatal association between PG and postpartum cardiomyopathy [41].

Most lesions occurred at the cesarean section incision site, emphasizing the significance of preventing traumatic lesions. Considering the presented cases, a cautious approach to vaginal delivery may be considered for pregnant patients with suspected PG, unless there is an absolute indication for cesarean section.

In terms of treatment modalities, 19 cases were managed with corticotherapy alone [5,6,18,20,22,27,30,31,32,35,36,40,41,42,44,50,51]. One case combined corticotherapy with surgery [21]. Eight cases received a combination of corticotherapy and cyclosporine, with one case also involving skin grafting [14,23,24,25,26,47,48,53]. Other treatment approaches included systemic corticotherapy and local tacrolimus [10]; systemic corticotherapy and dapsone [33,39]; a combination of corticotherapy, cyclosporine, and dapsone in two cases [29,34]; and a combination of corticotherapy, cyclosporine, and dapsone in Alani et al.’s case with mycophenolate mofetil [38]. Several cases required complex therapies, such as corticotherapy and azathioprine followed by vacuum-assisted closure and split-thickness skin graft [15]; negative pressure wound therapy and high-dose corticosteroid therapy [43,46]; corticotherapy and human immunoglobulin therapy; wound debridement, vacuum sealing negative pressure drainage, skin grafting, and hyperbaric oxygen therapy [4]; intravenous immunoglobulin alone [45]; and corticotherapy combined with intravenous immunoglobulin [49] or cyclosporine with skin grafting [53].

There is presently a lack of consensus on the treatment of PG, both during pregnancy and in non-pregnant patients, largely due to the rarity of the condition [19,63].

While the postpartum management and treatment of PG are relatively straightforward, as only the mother’s health and potential drug side effects need consideration, addressing PG during pregnancy becomes more intricate due to the necessity of considering the health of both the mother and the vulnerable, developing fetus. Most reviews advocate for systemic corticosteroids as the first-line treatment for PG, but these medications can have significant adverse effects on the fetus, including oral clefts, low birth weight (<2500 g), preterm birth (<37 weeks gestation), pre-eclampsia, and gestational diabetes mellitus. It is noteworthy that steroids are commonly used in the form of systemic treatment for many autoimmune illnesses in pregnant women with minimal fetal impact, and they are more extensively studied than any newly introduced treatment. The second most utilized drug in the treatment of PG is systemic cyclosporine, which is known for causing hypertension and nephrotoxicity. Therefore, these commonly used drugs for treating PG require careful consideration during pregnancy [19,63,64,65,66].

The use of intravenous immunoglobulin appears promising during pregnancy due to minimal side effects on the fetus [66,67].

Several other medications, including tacrolimus, azathioprine, minocycline, methotrexate, mycophenolate mofetil, sulfasalazine, and dapsone, have been used in combination or as monotherapy to treat this contentious condition. All of these medications have serious adverse effects when taken while pregnant [68].

Another category of drugs showing promising results and reduced side effects on the unborn child are TNF-α inhibitors, particularly infliximab, which has gathered substantial supporting data for its use in PG treatment, especially in cases associated with inflammatory bowel disease and those resistant to systemic corticosteroid therapy. Infliximab crosses the placenta after 30 weeks of gestation and can be detected in the baby’s serum postpartum for several months; however, studies suggest that there are no subsequent long-term immunological alterations in these children. Nonetheless, vaccination with live viruses should be avoided in these children for at least 6 months [66,69]. Wanberg et al. proposed in their 2023 case presentation the use of another TNF-α inhibitor, certolizumab; however, more data are needed regarding its benefits and potential side effects [70]. A 2019 systematic review of this category of treatment found no therapeutic difference in the results obtained by treating patients with PG with any of the three types of TNF-α inhibitors: infliximab, adalimumab, and etanercept [71].

Recent studies have shown that PG may be treated with biologic therapies such as IL-1 (interleukin-1) and IL-17 antagonists, like secukinumab and ixekizumab. Although reports of this particular monoclonal antibody therapy’s side effects are promising, more investigation is still required. This type of therapy is based on the reported peculiarities of this condition, such as the prevalence of neutrophils in patient skin biopsies. Brodalumab and anakira, anti-IL-17R medications, were also suggested as treatment strategies with a comparable rationale [68,71,72,73].

Of course, women with a personal or family history of PG are most at risk of developing it, although the precise risk is still unknown [74]. Second, due to the strong correlation with other autoimmune disorders, people with inflammatory bowel diseases (such as Crohn’s disease and ulcerative colitis), monoclonal gammopathies, hematological malignancies, and rheumatoid arthritis are at a heightened risk of developing PG lesions. According to case studies, seronegative arthritis, HIV, viral hepatitis, and pregnancy are among the other diseases that may predispose patients to develop PG [2,10,11]. Women who have a personal history of this condition need preventative precautions; vaginal delivery is the least risky and most recommended delivery mode in these situations. The criteria of the acronym PARACELSUS, with a significant clinical impact, are another helpful tool that Jockenhöfer et al. (2019) describe for accurately diagnosing PG lesions [75]. They outline some major, minor, and additional criteria with assigned values. A patient is most likely to have a PG lesion if the lesion presents a score of 10 or higher [66].

## 5. Conclusions

Hence, we posit that fostering interdisciplinary collaboration is imperative for the prompt identification and commencement of necessary therapeutic interventions in postpartum women afflicted by pyoderma gangrenosum. The submission of this particular case serves the overarching purpose of enhancing awareness surrounding the intricacies inherent in diagnosing and managing this challenging condition. This is especially pertinent in the context of our institutional setting, characterized by a unidisciplinary framework solely encompassing obstetric and gynecological services.

## Figures and Tables

**Table 1:** The cases reported in the literature.

Report (Author and Year of Publications)	Onset of the Disease	Site of the Lesion	Age (Years)	Recurrence	Associated Pathology	Treatment
Harlaand et al. (1993) [20]	Postpartum (29 weeks)	C-section	36	No	No	corticotherapy
Stone et al. (1996) [21]	Postpartum (29 weeks)	C-section	36	No	No	corticotherapy surgery
Steadman et al. (1998) [22]	Postpartum (37 weeks)	C-section	32	No	No	corticotherapy
Futami et al. (1998) [23]	Postpartum (vaginal birth)	Face, neck, upper arm, and thigh	32	Relapse	Ulcerative colitis	corticotherapy cyclosporine
Ronnau et al. (2000) [24]	Postpartum (not specified)	C-section	24	Yes (third episode)	Chronic type C hepatitis	corticotherapy cyclosporine
Skinner et al. (2006) [25]	Postpartum (39 weeks)	C-section	36	Yes After the vaginal delivery that occurred a year ago, she experienced a delayed healing perineal ulcer.	No	corticotherapy cyclosporine
Karim et al. (2006) [26]	Postpartum (37 weeks)	C-section	29	No	No	corticotherapy cyclosporine
Banga et al. (2007) [10]	Postpartum (29 weeks)	C-section	32	No	No	corticotherapy tacrolimus cream
Aoussar et al. (2007) [27]	During pregnancy (16 weeks) Postpartum (not specified)	On the torso and on the limbs	26	Yes After one year during pregnancy (30 weeks of gestation) and after two years in postpartum (on the right arm—intramuscular injection site).	Takayasu arteritis, type B hepatitis	corticotherapy
Sanz-Munoz et al. (2008) [5]	During pregnancy postpartum (not specified)	Anterior side of the left leg 4 days earlier C-section	30	No	No	corticotherapy
Wiersma et al. (2009) [28]	Postpartum (not specified)	C-section	31	No	No	corticotherapy
Pauser et al. (2009) [29]	Postpartum (not specified)	C-section	39	No	No	corticotherapy cyclosporine dapsone
Wierzbicka-Hainaut et al. (2010) [30]	During pregnancy (third trimester) Postpartum (37 weeks)	Left calf C-section	25	Yes During her first and second pregnancy.	No	corticotherapy
Kikuchi et al. (2010) [31]	Postpartum (not specified)	C-section	28	No	No	corticotherapy
Muresan et al. (2010) [32]	Postpartum (term)	C-section	27	Yes	No	corticotherapy
Amin et al. (2014) [33]	Postpartum (32 weeks)	C-section	32	Yes Third recurrence, after every one of the three C-sections.	No	corticotherapy dapsone
Aydin et al. (2015) [15]	Postpartum (37 weeks)	C-section	32	Yes Previous C-section and left hand at the site of intravenous catheterization.	No	corticotherapy azathioprine—unresponsive vacuum-assisted closure split-thickness skin graft
Radhika et al. (2015) [34]	Postpartum (term)	C-section	22	Yes After an intramuscular injection on her buttock.	No	corticotherapy cyclosporin dapsone
Simmons et al. (2015) [35]	Postpartum (27 weeks)	C-section	43	No	No	corticotherapy
Cokan et al. (2016) [36]	Postpartum (not specified abruptio placentae)	C-section Left breast	23	No	No	corticotherapy
Park et al. (2016) [14]	Postpartum (term)	C-section	33	No	No (antiphospholipid antigen—weakly positive)	corticotherapy cyclosporine
Nonaka et al. (2016) [37]	Postpartum (36 weeks)	C-section	39	No	Antiphospholipid syndrome (lupus anticoagulant weakly positive but with 3 spontaneous abortions)	corticotherapy minocycline hydrochloride
Alani et al. (2016) [38]	Postpartum (not specified)	Episiotomy	29	No	Hepatitis C	Corticotherapy cyclosporine mycophenolate mofetil dapsone
Hilton et al. (2017) [39]	Postpartum (not specified)	C-section	28	No	No	corticotherapy dapsone
Satoh et al. (2018) [40]	Postpartum (33 weeks)	C-section	27	Yes First episode after appendectomy 10 years previously.	No	corticotherapy
Diallo et al. (2017) [18]	Postpartum (not specified)	C-section	25	No	No	corticotherapy
Naciri et al. (2018) [41]	Postpartum (term)	C-section	23	Yes Abscess on her left breast prior to pregnancy	Peripartum/Postpartum cardiomyopathy	corticotherapy
Murata et al. (2019) [42]	Postpartum (37 weeks)	C-section	29	Yes	No	corticotherapy
	Postpartum (33 weeks)	C-section	27	Yes	No	corticotherapy
Foessleitner et al. (2019) [43]	Postpartum (30 weeks)	C-section	34	No	No	negative pressure wound therapy high-dose corticosteroids therapy
Shen et al. (2019) [4]	Postpartum (>7 months of gestation)	C-section	32	No	No	corticotherapy human immunoglobulin wound debridement vacuum sealing negative pressure drainage skin grafting hyperbaric oxygen therapy
van Donkelaar et al. (2020) [44]	Postpartum (32 weeks)	C-section	21	No	No	corticotherapy
Gunduz et al. (2020) [45]	Postpartum (not specified)	C-section Bilateral gluteal regions post NSAIDs administration	32	No	No	intravenous immunoglobulin
Zolper et al. (2021) [46]	Postpartum (not specified)	C-section	28	Yes First episode (post-vaginal delivery)	No	corticotherapy cyclosporine incisional negative pressure wound therapy
Moutos et al. (2021) [47]	Postpartum (28 weeks)	C-section	23	No	No	corticotherapy cyclosporine
Ghumra et al. (2021) [48]	Postpartum (31 weeks)	C-section	35	Yes 14 years prior, skin grafts for ulcers on her left lower leg	No	corticotherapy cyclosporine
Luo et al. (2021) [49]	Postpartum (not specified)	C-section	38	No	Hepatitis B	corticotherapy intravenous immunoglobulin
Rao et al. (2021) [6]	Postpartum (not specified)	Episiotomy	28	No	No	corticotherapy
Aziret et al. (2021) [50]	Postpartum (term)	C-section	36	No	No	corticotherapy
Ishizaki et al. (2022) [51]	Postpartum (36 weeks)	C-section	34	No	Thrombocytopenia early in pregnancy	corticotherapy
Bal et al. (2022) [52]	Postpartum (not specified abruptio placentae)	C-section	25	Yes 2 years prior—injection abscess right gluteal region healed with a large scar	No	corticotherapy
Lyons et al. (2023) [53]	Postpartum	Right breast	32	No	No	corticotherapy cyclosporine skin graft

## Data Availability

The data used to support the findings of this study are available upon request from the authors.

## References

[1] Sarwar S., Sajid F., Wasim A.U., Waleed M.S., Thada P.K. (2023). Pyoderma Gangrenosum Precipitated by Breast Engorgement Following Lactation Discontinuation: A Rare Case Report. Cureus.

[2] Gameiro A., Pereira N., Cardoso J.C., Gonçalo M. (2015). Pyoderma gangrenosum: Challenges and solutions. Clin. Cosmet. Investig. Dermatol..

[3] George C., Deroide F., Rustin M. (2019). Pyoderma gangrenosum—A guide to diagnosis and management. Clin. Med. (Lond.).

[4] Shen J., Zhang W., Jiang X. (2019). Pyoderma gangrenosum after cesarean section treated with skin graft: A case report. Medicine.

[5] Sanz-Muñoz C., Martínez-Morán C., Miranda-Romero A. (2008). Pyoderma Gangrenosum Following Cesarean Delivery. Actas Dermosifiliogr..

[6] Rao S., Jain V., Bhattacharjee R., Vishwajeet V., Thakur G. (2021). Pyoderma gangrenosum at an episiotomy site in successive pregnancies: A case report. Case Rep. Womens Health.

[7] Su W.P., Davis M.D., Weenig R.H., Powell F.C., Perry H.O. (2004). Pyoderma gangrenosum: Clinicopathologic correlation and proposed diagnostic criteria. Int. J. Dermatol..

[8] Bennett M.L., Jackson J.M., Jorizzo J.L., Fleischer A.B., White W.L., Callen J.P. (2000). Pyoderma gangrenosum. A comparison of typical and atypical forms with an emphasis on time to remission. Case review of 86 patients from 2 institutions. Medicine.

[9] Suvirya S., Pathania S., Singhai A. (2019). A case of bullous pyoderma gangrenosum. BMJ Case Rep..

[10] Banga F., Schuitemaker N., Meijer P. (2006). Pyoderma gangrenosum after caesarean section: A case report. Reprod. Health.

[11] Ahn C., Negus D., Huang W. (2018). Pyoderma gangrenosum: A review of pathogenesis and treatment. Expert. Rev. Clin. Immunol..

[12] Rodríguez-Zúñiga M.J.M., Heath M.S., Gontijo J.R.V., Ortega-Loayza A.G. (2019). Pyoderma gangrenosum: A review with special emphasis on Latin America literature. An. Bras. Dermatol..

[13] Teagle A., Hargest R. (2014). Management of pyoderma gangrenosum. JR Soc. Med..

[14] Park J.Y., Lee J., Park J.S., Jun J.K. (2016). Successful vaginal birth after prior cesarean section in a patient with pyoderma gangrenosum. Obstet. Gynecol. Sci..

[15] Aydin S., Aydın Ç.A., Uğurlucan F.G., Yaşa C., Dural Ö. (2015). Recurrent pyoderma gangrenosum after cesarean delivery successfully treated with vacuum-assisted closure and split thickness skin graft: A case report. J. Obstet. Gynaecol. Res..

[16] Ahronowitz I., Harp J., Shinkai K. (2012). Etiology and Management of Pyoderma Gangrenosum. Am. J. Clin. Dermatol..

[17] Rashid R.M. (2008). Seat belt pyoderma gangrenosum: Minor pressure as a causative factor. J. Eur. Acad. Dermatol. Venereol..

[18] Diallo M., Diop A., Diatta B.A., Diadie S., Ndiaye M., Ndiaye M.T., Seck B., Deh A., Diop K. (2017). Post-Partum Pyoderma Gangenosum Following a Cesarean Section. Dermatol. Case Rep..

[19] Steele R.B., Nugent W.H., Braswell S.F., Frisch S., Ferrell J., Ortega-Loayza A.G. (2016). Pyoderma gangrenosum and pregnancy: An example of abnormal inflammation and challenging treatment. Br. J. Dermatol..

[20] Harland C.C., Jaffe W., Holden C.A., Ross L.D. (1993). Pyoderma gangrenosum complicating caesarean section. J. Obstet. Gynaecol..

[21] Stone N., Harland C., Ross L., Holden C. (1996). Pyoderma gangrenosum complicating caesarian section. Clin. Exp. Dermatol..

[22] Steadman U.A., Brennan T.E., Daman L.A., Curry S.L. (1998). Pyoderma gangrenosum following cesarean delivery. Obstet. Gynecol..

[23] Futami H., Kodaira M., Furuta T., Hanai H., Kaneko E. (1998). Pyoderma gangrenosum complicating ulcerative colitis: Successful treatment with methylprednisolone pulse therapy and cyclosporine. J. Gastroenterol..

[24] Ronnau A.C., von Schmiedeberg S. (2000). Pyoderma gangrenosum after cesarean delivery. Am. J. Obstet. Gynecol..

[25] Skinner A.M., Mills S.M. (2006). Management of a chronic wound secondary to pyoderma gangrenosum following uncomplicated lower segment Caesarean section incision. Aust. N. Z. J. Obstet. Gynaecol..

[26] Karim A.A., Ahmed N., Salman T.A., Craven N.M. (2006). Pyoderma gangrenosum in pregnancy. J. Obstet. Gynaecol..

[27] Aoussar A., Ismaïli N., Berbich L., Tazi Mezalek Z., Aït Ourhrouil M., Senouci K., Mansouri F., Hassam B. (2007). Pyoderma gangrenosum révélant une artérite de Takayasu [Pyoderma gangrenosum revealing Takayasu’s arteritis]. Ann. Dermatol. Venereol..

[28] Wiersma I.C., Braams-Lisman B.A., Mekkes J.R. (2009). Pyoderma gangraenosum na een sectio caesarea [Pyoderma gangrenosum after a cesarean section]. Ned. Tijdschr. Geneeskd..

[29] Pauser S., Goerge T., Eickelmann M., Markus G., Luger T., Steinoff M. (2009). Pyoderma Gangrenosum After Cesarean Delivery. Clin. Med. Insights Dermatol..

[30] Wierzbicka-Hainaut E., Le Naoures H., Bonneric-Malthieu F., Debiais P., Levillain P., Pierre F., Guillet G. (2010). Recurring Pyoderma gangrenosum in pregnancy. Ann. Dermatol. Venereol..

[31] Kikuchi N., Hanami Y., Miura T., Kawakami Y., Satoh M., Ohtsuka M., Yamamoto T. (2010). Pyoderma gangrenosum following surgical procedures. Int. J. Dermatol..

[32] Muresan M. (2011). Successful relactation—A case history. Breastfeed. Med..

[33] Amin S.V., Bajapai N., Pai A., Bharatnur S., Hebbar S. (2014). Pyoderma gangrenosum in two successive pregnancies complicating caesarean wound. Case Rep. Obstet. Gynecol..

[34] Radhika A.G., Singal A., Radhakrishnan G., Singh S. (2015). Pyoderma gangrenosum following a routine caesarean section: Pseudo-infection in a caesarean wound. Qatar Med. J..

[35] Simmons P., Artis A., Dwiggins M., Gross T., LoCoco S., Farell J. (2015). 31: A rare case of pyoderma gangrenosum and the effects of pathergy in a postpartum patient. AJOG.

[36] Cokan A., Dovnik A., Žebeljan I., Mujezinović F., Bujas T., Marko P.B. (2016). Pyoderma gangrenosum in a caesarean wound following caesarean section with late occurrence of pyoderma gangrenosum of the breast. Eur. J. Obstet. Gynecol. Reprod. Biol..

[37] Nonaka T., Yoshida K., Yamaguchi M., Aizawa A., Fujiwara H., Enomoto T., Takakuwa K. (2016). Case with pyoderma gangrenosum abruptly emerging around the wound of cesarean section for placenta previa with placenta accrete. J. Obstet. Gynaecol. Res..

[38] Alani A., Sadlier M., Ramsay B., Ahmad K. (2016). Pyoderma gangrenosum induced by episiotomy. BMJ Case Rep..

[39] Hilton R., Berryman J., Handoyo K. (2017). Pyoderma Gangrenosum Masquerading as Necrotizing Fasciitis: Stepping Away from Cognitive Shortcuts. Eur. J. Case Rep. Intern. Med..

[40] Satoh M., Hiraiwa T., Yamamoto T. (2018). Recurrent pyoderma gangrenosum developed after a cesarean section with a 10-year interval. Int. J. Dermatol..

[41] Naciri I., Meziane M., Benzekri L., Ghaouti M., Senouci K., Hassam B. (2018). Pyoderma gangrenosum récidivant du post-partum et cardiomyopathie fatale [ecurrent postpartum pyoderma gangrenosum and fatal cardiomyopathy]. Ann. Dermatol. Venereol..

[42] Murata T., Kyozuka H., Fukuda T., Hiraiwa T., Yamaguchi A., Fujimori K. (2019). Incisional pyoderma gangrenosum after caesarean section: Two case reports. Case Rep. Womens Health.

[43] Foessleitner P., Just U., Kiss H., Farr A. (2019). Challenge of diagnosing pyoderma gangrenosum after caesarean section. BMJ Case Rep..

[44] van Donkelaar C.E., de Haan J.M.H., Lange J.F.M., de Vries M., Horváth B. (2020). Pseudo-wound infection after a caesarean section: Case report of unrecognized Pyoderma Gangrenosum. Int. J. Surg. Case Rep..

[45] Gündüz K., Gülbaşaran F., Hasdemir P.S., Temiz P., Inanır I. (2020). Successful treatment of severe refractory post-cesarean pyoderma gangrenosum with intravenous immunoglobulin. Dermatol. Ther..

[46] Zolper E.G., Harbour P.W., Dekker P.K., Schwitzer J.A., Viramontes A., Evans K.K. (2021). Post-Cesarean Section Pyoderma Gangrenosum Presenting with Vasopressor-dependent Shock: Long-term Follow-up after Delayed Primary Closure. Plast. Reconstr. Surg. Glob. Open.

[47] Moutos C.P., Hoyer P., Kelly B., Saad A.F. (2021). Pyoderma gangrenosum after cesarean delivery. Am. J. Obstet. Gynecol..

[48] Ghumra W., Gold A., Azurdia R.M. (2021). Pyoderma gangrenosum following an unplanned caesarean section: A patient revisited. BMJ Case Rep..

[49] Luo H., Bian H., Sun C., Zheng S., Xiong B., Huang Z., Liu Z., Ma L., Li H., Chen H. (2021). Pyoderma gangrenosum secondary to caesarean section treated with negative pressure wound therapy and skin graft. Dermatol. Ther..

[50] Aziret M., Kara Ş., Yaldız M., Köse N., Aşıkuzunoğlu F., Cevrioğlu A.S. (2021). An extensive pyoderma gangrenosum mimicking necrotizing fasciitis: An unusual case report. Int. J. Surg. Case Rep..

[51] Ishizaki R., Yamamoto M., Yamamoto T. (2022). Bullous Pyoderma Gangrenosum Occurring on a Cesarean Section Scar. Indian J. Dermatol..

[52] Bal H., Subramanian S., Sharma Y.K., Lal S. (2022). A Case of Post-caesarean Pyoderma Gangrenosum. J. Obstet. Gynaecol. India.

[53] Lyons D., Sexton F., Martin-Smith J., Raghallaigh S.N. (2023). Postpartum pyoderma gangrenosum following lactation-induced mastitis and abscess incision. JAAD Case Rep..

[54] Ruocco E., Sangiuliano S., Gravina A.G., Miranda A., Nicoletti G. (2009). Pyoderma gangrenosum: An updated review. J. Eur. Acad. Dermatol. Venereol..

[55] Keskin M., Tosun Z., Ucar C., Savaci N. (2006). Pyoderma gangrenosum in a battered child. Ann. Plast. Surg..

[56] Reddy K., Brightman L., Venna S. (2008). Pyoderma gangrenosum with pathergy in a pregnant patient without associated systemic disease. Cutis.

[57] Maverakis E., Ma C., Shinkai K., Fiorentino D., Callen J.P., Wollina U., Marzano A.V., Wallach D., Kim K., Schadt C. (2018). Diagnostic Criteria of Ulcerative Pyoderma Gangrenosum: A Delphi Consensus of International Experts. JAMA Dermatol..

[58] Cox A., Tomlin K. (2022). A noninfectious “infection” after cesarian delivery. Contemp. OB/GYN.

[59] Weenig R.H., Davis M.D., Dahl P.R., Su W.P. (2002). Skin ulcers misdiagnosed as pyoderma gangrenosum. N. Engl. J. Med..

[60] Ashchyan H.J., Nelson C.A., Stephen S., James W.D., Micheletti R.G., Rosenbach M. (2018). Neutrophilic dermatoses: Pyoderma gangrenosum and other bowel- and arthritis-associated neutrophilic dermatoses. J. Am. Acad. Dermatol..

[61] Morgenstjerne-Schwenck L.E.T., Knudsen J.T., Prasad S.C. (2021). Efficacy and safety of skin grafting in treatment of vasculitic ulcer and pyoderma gangrenosum-A systematic review. Wound Repair. Regen..

[62] Goto H., Okada Y., Watanabe S., Danno K., Yamamoto S., Ishiura R., Nakayama Y., Mitsui K., Ueki A., Nakai Y. (2021). Successful Treatment of Ulcerative-Type Pyoderma Gangrenosum with a Combination Therapy of Oral Prednisolone, Vacuum-Assisted Closure, and Skin Grafting. Case Rep. Dermatol..

[63] Stiegler J.D., Lucas C.T., Sami N. (2017). Pyoderma gangrenosum in pregnancy successfully treated with infliximab and prednisone. JAAD Case Rep..

[64] Ohmaru-Nakanishi T., Goto H., Maehara M., Oishi H., Ueoka Y. (2019). Perineal pyoderma gangrenosum in pregnancy: A case report. Case Rep. Womens Health.

[65] Bandoli G., Palmsten K., Forbess Smith C.J., Chambers C.D. (2017). A Review of Systemic Corticosteroid Use in Pregnancy and the Risk of Select Pregnancy and Birth Outcomes. Rheum. Dis. Clin. N. Am..

[66] Vigl K., Posch C., Richter L., Monshi B., Rappersberger K. (2016). Pyoderma gangrenosum during pregnancy—Treatment options revisited. J. Eur. Acad. Dermatol. Venereol..

[67] Erfurt-Berge C., Herbst C., Schuler G., Bauerschmitz J. (2012). Successful treatment of pyoderma gangrenosum with intravenous immunoglobulins during pregnancy. J. Cutan. Med. Surg..

[68] Kao A.S., King A.D., Bardhi R., Daveluy S. (2023). Targeted therapy with ixekizumab in pyoderma gangrenosum: A case series and a literature overview. JAAD Case Rep..

[69] Djokanovic N., Klieger-Grossmann C., Pupco A., Koren G. (2011). Safety of infliximab use during pregnancy. Reprod. Toxicol..

[70] Wanberg L.J., Fletcher K.M., Goldfarb N., Alavi A. (2023). Treatment of pyoderma gangrenosum in pregnancy with certolizumab pegol. JEADV Clin. Prac..

[71] Ben Abdallah H., Fogh K., Bech R. (2019). Pyoderma gangrenosum and tumour necrosis factor alpha inhibitors: A semi-systematic review. Int. Wound J..

[72] O’Connor C., Gallagher C., Hollywood A., Paul L., O’Connell M. (2021). Anakinra for recalcitrant pyoderma gangrenosum. Clin. Exp. Dermatol..

[73] Petty A.J., Whitley M.J., Balaban A., Ellington K., Marano A.L. (2020). Pyoderma gangrenosum induced by secukinumab in a patient with psoriasis successfully treated with ustekinumab. JAAD Case Rep..

[74] Xia F.D., Liu K., Lockwood S., Butler D., Tsiaras W.G., Joyce C., Mostaghimi A. (2018). Risk of developing pyoderma gangrenosum after procedures in patients with a known history of pyoderma gangrenosum-A retrospective analysis. J. Am. Acad. Dermatol..

[75] Jockenhöfer F., Wollina U., Salva K.A., Benson S., Dissemond J. (2019). The PARACELSUS score: A novel diagnostic tool for pyoderma gangrenosum. Br. J. Dermatol..

